# Supplementary material for: Large Scale Vat-Photopolymerization of Investment Casting Master Patterns: The Total Solution
Source: Polymers (Basel). 2022 Oct 29;14(21):4593. doi: 10.3390/polym14214593 (PMC9656478; doi:10.3390/polym14214593)
Supplement: Supplementary file 1 [file polymers-14-04593-s001.zip › polymers-1939469-supplementary.pdf]

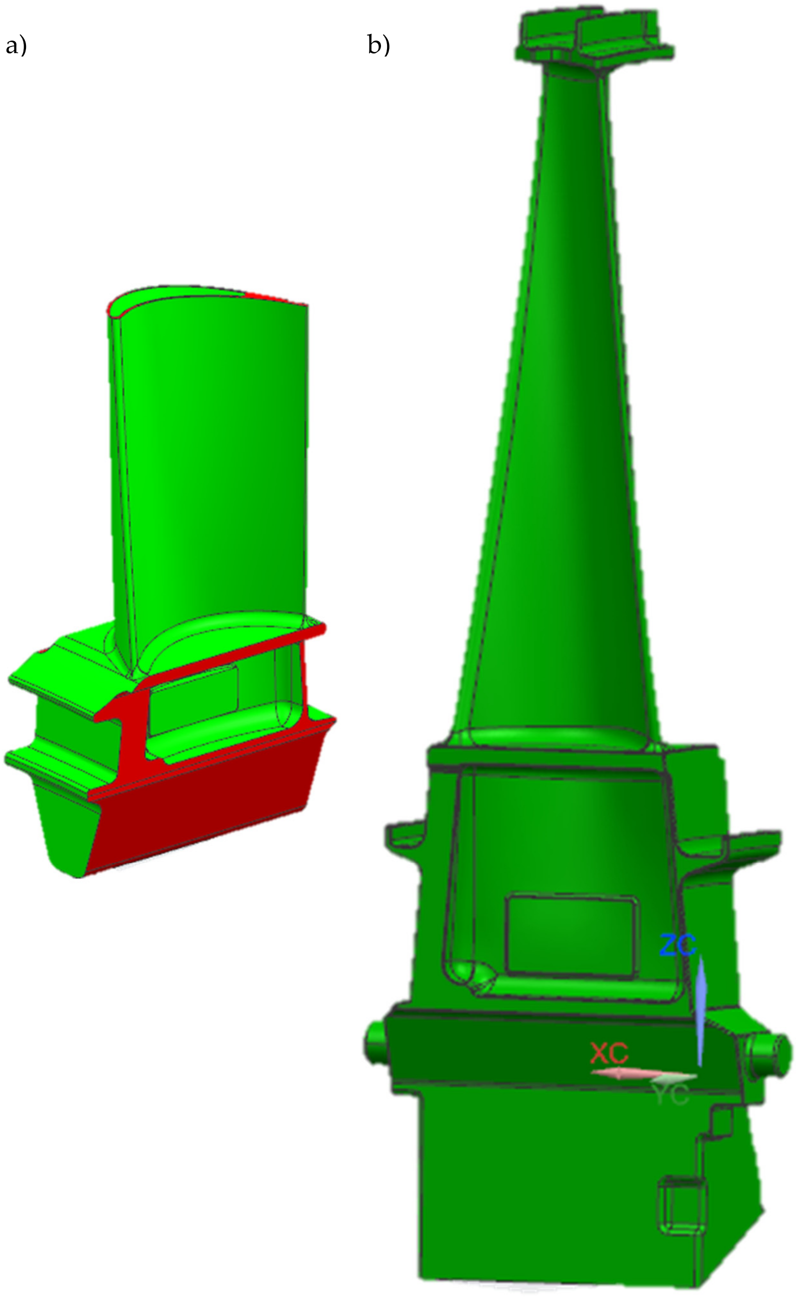

Figure S1 Two typical solid blade geometries for (a) Dimensional measurements, and (b) Burnout experiments.

Table S1 Dimensional deviations the turbine blade printed using A40W50 mixture over a 21-day period. Part is in its green (unpost-cured) condition.

		A40W50			
	#Point	Day 0	Day 7	Day 14	Day 21
Section 1	1	-85	-200	-55	-62
	2	-29	-53	35	46
	3	15	-35	180	168
	4	1	-47	264	222
	5	-18	-99	44	36
	6	108	131	169	189
	7	84	57	74	101
	8	116	100	46	87
	9	56	95	24	64
Section 2	10	-73	-111	-119	-105
	11	71	15	172	178
	12	16	20	81	108
	13	126	127	265	247
	14	241	220	317	232
	15	-8	-30	-17	-41
	16	-237	-276	-225	-263
	17	22	-13	-29	-3
	18	112	50	44	44
	19	-11	-60	38	41
	Average (+)	69.40	90.56	125.21	125.93
	Average (-)	-65.86	-92.40	-89.00	-94.80

Table S2 Dimensional deviations the turbine blade printed using A40W50 mixture over a 21-day period. Part is in its post-cured (A40W50-PC) condition.

		A40W50-PC				
	#Point	Before PC	Day 0	Day 7	Day 14	Day 21
Section 1	1	-164	-278	-249	-149	-191
	2	-18	-90	-67	6	-1
	3	-60	-61	-59	-57	-55
	4	-56	-77	-69	-41	-66
	5	-46	-130	-132	-67	-91
	6	87	155	135	84	107
	7	69	101	55	36	60
	8	133	138	100	79	135
	9	139	98	90	129	161
Section 2	10	-108	-169	-161	-99	-133
	11	-15	-6	-5	30	-8
	12	-13	3	6	17	13
	13	93	142	114	117	100
	14	228	237	203	210	215
	15	-51	-44	-43	-37	-43
	16	-250	-292	-289	-296	-286
	17	34	-19	-25	-16	8
	18	94	10	31	60	63
	19	-53	-68	-66	-28	-25
	Average (+)	109.63	110.50	91.75	76.80	95.78
	Average (-)	-75.82	-112.18	-105.91	-87.78	-89.90
